# Vaccination Uptake and Pandemic-Related Healthcare Experiences Among People Living with HIV in Romania: A Cross-Sectional Study

**DOI:** 10.3390/vaccines14090751

**Published:** 2026-08-28

**Authors:** Adrian Gabriel Marinescu, Aida-Isabela Adamescu, Cătălin Tilișcan, Daniela Pițigoi, Sebastian Ciobanu, Laurențiu Mihăița Stratan, Carmen Cristina Vasile, Victor Daniel Miron, Mariana Mardarescu, Dalila Toma, Andreea Iuliana Ciobanu, Victoria Aramă, Ștefan Sorin Aramă

**Affiliations:** 1 Faculty of Medicine, Department II, Discipline of Infectious Diseases II, Carol Davila University of Medicine and Pharmacy, 37 Dionisie Lupu Street, 020021 Bucharest, Romania; 2 Faculty of Dental Medicine, Department II, Discipline of Pathophysiology and Immunology, Carol Davila University of Medicine and Pharmacy, 37 Dionisie Lupu Street, 020021 Bucharest, Romania; 3 Department of Infectious Diseases, National Institute of Infectious Diseases “Prof. Dr. Matei Bals”, 020021 Bucharest, Romania; 4 Department of Radiology and Medical Imaging, Emergency University Hospital, 050098 Bucharest, Romania

**Keywords:** people living with HIV (PLWH), vaccination uptake, vaccination hesitancy, COVID-19 pandemic, preventive healthcare, healthcare access, vaccine perceptions, Romania

## Abstract

**Background:** The COVID-19 pandemic exposed major vulnerabilities in healthcare accessibility and may have influenced preventive attitudes among people living with HIV (PLWH). However, the relationship between pandemic-related healthcare experiences and vaccination uptake remains insufficiently understood. **Materials:** A cross-sectional survey was conducted among 203 adults living with HIV receiving follow-up care in Romania between October 2025 and March 2026. The questionnaire evaluated vaccination uptake, vaccine-related perceptions, healthcare information sources, and pandemic-related experiences. Multivariable logistic regression analysis was performed to identify factors independently associated with vaccination uptake. **Results:** Only 38.9% of participants reported receiving at least one vaccine within the previous three years. The main barriers to vaccination were perceived lack of necessity, lack of physician recommendation, fear of adverse effects, and lack of trust. Multivariable logistic regression identified positive vaccine-related beliefs (OR = 0.454, 95% CI: 0.235–0.876, *p* = 0.019) and CD4+ T-cell count above 500 cells/mm^3^ (OR = 0.448, 95% CI: 0.232–0.867, *p* = 0.017) as independently associated with lower odds of being non-vaccinated. More than half of participants (52.7%) reported at least one pandemic-related disruption or negative health impact. The pandemic-related negative impact composite was not significantly associated with vaccination status (*p* = 0.209), whereas confirmed COVID-19 infection was more frequent among vaccinated participants than among non-vaccinated participants (43.0% vs. 28.2%, *p* = 0.030). Pandemic-related negative impact was associated with a greater demand for physician-provided information (*p* = 0.011). **Conclusions:** Pandemic-related healthcare experiences were associated with greater demand for physician-provided preventive information, while confirmed COVID-19 infection was more frequent among vaccinated participants. Positive vaccine-related beliefs and higher CD4+ T-cell counts were independently associated with vaccination uptake, while lack of physician recommendation remained a major barrier to vaccination. These findings emphasize the critical role of physicians in improving vaccination coverage among people living with HIV in the post-pandemic era.

## 1. Introduction

The COVID-19 pandemic exposed major vulnerabilities in healthcare systems worldwide and substantially disrupted both preventive and curative medical services. Many patients delayed or avoided medical follow-up because of fear of infection, while healthcare facilities experienced significant reductions in routine service utilization [1]. These disruptions disproportionately affected individuals requiring continuous long-term medical care, including patients with chronic diseases and multimorbidity. Beyond its direct infectious burden, the pandemic also altered healthcare-seeking behaviour and underscored the importance of resilient healthcare systems, continuity of care, and preventive medicine strategies during global public health crises [2]. The pandemic also altered infection-related complications in vulnerable populations, emphasizing the need for comprehensive preventive and monitoring strategies beyond the acute management of COVID-19 [3].

People living with HIV (PLWH) represent a particularly vulnerable population requiring lifelong medical care, regular monitoring, and sustained preventive health interventions. Advances in antiretroviral therapy have transformed HIV infection into a chronic, manageable condition; however, long-term disease management remains highly dependent on uninterrupted access to healthcare services and sustained patient engagement [4]. During the pandemic, healthcare systems redirected resources towards acute COVID-19 management, reducing access to routine consultations and preventive services for patients with chronic conditions [5,6]. In this context, maintaining preventive healthcare measures among PLWH, including vaccination uptake and physician-guided counselling, became increasingly important during and after the pandemic period [7]. In addition to chronic immune dysfunction, PLWH may remain vulnerable to infectious complications and adverse health outcomes, particularly when barriers to healthcare access and continuity of care are present [8]. HIV infection has been associated with accelerated biological aging processes and the development of age-related comorbidities, emphasizing the need for sustained preventive healthcare interventions throughout long-term follow-up [9].

The pandemic has also reshaped prophylactic health behaviours and perceptions at both individual and community levels. Engagement in preventive measures is strongly influenced by individual beliefs, perceived susceptibility, perceived benefits, and trust in healthcare recommendations, highlighting the importance of effective physician-led communication during public health emergencies [10]. Recent studies have shown that healthcare utilization and preventive health screening remained below pre-pandemic levels even after the acute phase of COVID-19, suggesting persistent disruption of preventive healthcare services [11].

Vaccination represents one of the most effective prophylactic strategies for reducing infectious disease-related morbidity, hospitalization, and mortality among people living with HIV. Owing to persistent immune dysregulation and increased susceptibility to opportunistic and vaccine-preventable infections, international guidelines strongly recommend routine immunization in this population [12,13]. Nevertheless, vaccine uptake among PLWH remains suboptimal worldwide. Multiple barriers contribute to low vaccination coverage, including stigma, lack of trust in healthcare systems, and insufficient physician recommendation [14]. Furthermore, HIV-related immune dysfunction increases susceptibility to several infections that are preventable by immunization, further emphasizing the importance of vaccination in this population [12,15,16,17].

Vaccine hesitancy among PLWH is influenced by multiple factors, including concerns regarding vaccine safety, fear of adverse effects, misinformation, medical mistrust, and inadequate physician communication. Trust in healthcare providers has consistently been identified as a major determinant of vaccine acceptance, while patient-centred communication may improve confidence in vaccination and measures that could be used to prevent the development of certain diseases among PLWH [18]. Recent evidence also suggests that misinformation and conspiracy-related beliefs may influence vaccine confidence among vulnerable populations, including PLWH. These findings highlight the importance of proactive physician-led education and targeted public health communication strategies aimed at improving vaccine confidence and preventive engagement [19]. Pandemic-related experiences may have influenced vaccination perceptions among PLWH. At the same time, disruptions in healthcare access, interruptions in HIV-related services, and widespread exposure to conflicting medical information may have altered trust in vaccines and medical care, thereby influencing attitudes towards medical systems [20,21].

Although several studies have evaluated COVID-19 vaccine acceptance among PLWH, little is known about the broader impact of the pandemic on vaccination behaviours, preventive healthcare attitudes, and healthcare engagement in this population. Addressing this knowledge gap is essential for developing targeted interventions aimed at improving vaccine uptake and strengthening long-term preventive care among PLWH [22,23,24]. In Romania, adults living with HIV are recommended to receive several vaccines according to national and international guidelines, including influenza, pneumococcal, hepatitis A, hepatitis B, meningococcal, HPV, varicella, and COVID-19 vaccines, depending on age, immunological status, and individual risk factors. Furthermore, recent legislative changes have expanded access to vaccination through reimbursement programs for selected vaccines in high-risk populations, including individuals with chronic medical conditions. Despite these efforts, data regarding vaccination uptake among PLWH in Romania remain limited [25]. The present study aimed to evaluate vaccination uptake, vaccine-related perceptions, healthcare information sources, and the impact of the COVID-19 pandemic on healthcare behaviours among PLWH. We also aimed to identify factors associated with vaccination uptake.

## 2. Materials and Methods

A single-centre cross-sectional survey was conducted among adults living with HIV using a structured questionnaire. Participants were recruited from individuals receiving follow-up care at the National Institute of Infectious Diseases “Prof. Dr. Matei Bals” in Bucharest, Romania, the largest infectious diseases hospital in the country, designated as a reference centre for the management of HIV/AIDS and COVID-19 cases and other public health alerts in Romania [26]. The study centre provides care to more than 4000 PLWH and represents one of the largest HIV care centres in Romania. However, aggregate demographic data for the entire clinical population were not available for the comparison. Data collection took place over a six-month period, from October 2025 through March 2026, and included only patients aged 18 years or older. A sample size calculation was performed prior to study initiation, indicating that a minimum of 200 participants would be required to ensure adequate statistical power. Consecutive eligible participants were subsequently enrolled until the target sample size was reached.

Individuals were considered eligible if they had a documented diagnosis of HIV infection and were under regular follow-up. Recruitment was carried out among patients attending scheduled visits at the outpatient day-care facility when they underwent routine medical assessments and received antiretroviral therapy. All eligible patients presenting during the study duration were invited to participate using a consecutive inclusion approach. A total of 221 eligible individuals were approached for participation, of whom 203 agreed to participate and were enrolled in the study, corresponding to a participation rate of 91.9%, while 18 individuals (8.1%) declined participation. Only participants who completed the questionnaire in its entirety were included in the final analysis, and no imputation was performed for questionnaire-derived variables. Clinical variables, including the last documented HIV viral load, CD4+ T-cell count, and ART adherence, were obtained separately from medical records. Although all included participants had complete questionnaires, these clinical variables were not available or documented for all participants at the time of data extraction. Missing medical-record-derived data were not considered an exclusion criterion. Analyses involving these variables were performed using the available-case analysis, without imputation, and missing observations are explicitly reported in Table 1. Individuals were not included in the analysis if they refused enrolment, failed to provide written informed consent, or submitted questionnaires with missing or incomplete responses.

A study-specific questionnaire was developed and administered to all enrolled participants. The complete questionnaire used for data collection is provided as Supplementary Material File S1. The questionnaire collected information on demographic characteristics, self-reported HIV-related history, personal history of past infections, and vaccination history over the previous 3 years, including pneumococcal, influenza, meningococcal, hepatitis A, hepatitis B, and other vaccinations. Another part of the questionnaire was about vaccine-related perceptions, sources of vaccine-related information, and the perceived impact of the COVID-19 pandemic on healthcare access and vaccination behaviour.

Clinical and immunological data, including HIV viral load, CD4+ T-cell count, treatment adherence, comorbidities, and previous opportunistic infections, were obtained from medical records and incorporated into the analysis. Variables included HIV viral load status, CD4+ T-cell count category (<200 cells/mm^3^, 200–350 cells/mm^3^, 351–500 cells/mm^3^, and >500 cells/mm^3^), duration since HIV infection, duration of antiretroviral treatment, treatment adherence, and previous AIDS-defining conditions or opportunistic infections. Additional data regarding comorbidities were also obtained from medical records. Information regarding the impact of the COVID-19 pandemic on access to healthcare services and antiretroviral therapy was collected through the questionnaire.

The questionnaire was developed based on the study objectives and on previously published literature addressing vaccination uptake, vaccine hesitancy, and healthcare practices among PLWH. The study protocol received approval from the Ethics Committee of the National Institute of Infectious Diseases “Prof. Dr. Matei Bals,” protocol number: C14163/09.12.2025. All study procedures were conducted in accordance with the principles outlined in the Declaration of Helsinki, and written informed consent was obtained from every participant prior to enrolment.

### Statistical Analysis

Data analysis was performed using IBM SPSS Statistics version 20.0 (IBM Corp., Armonk, NY, USA). Continuous variables were evaluated for normality using the Kolmogorov–Smirnov test and were reported as means with standard deviations (SD) or as medians with interquartile ranges (IQR), depending on data distribution. Categorical variables were summarized using frequencies and percentages. To assess overall vaccine uptake, a composite vaccination variable was generated based on self-reported receipt of at least one recommended vaccine during the previous three years, including influenza, pneumococcal, meningococcal, hepatitis A, hepatitis B, varicella, HPV, COVID-19, or other vaccines. Participants reporting receipt of at least one vaccine were classified as vaccinated, whereas those reporting no vaccination were classified as non-vaccinated.

Group comparisons according to vaccination status were conducted using Pearson’s chi-square test or Fisher’s exact test for categorical variables and the Mann–Whitney U test for continuous variables when appropriate. For clinical variables with missing observations, statistical analyses were performed using available cases only, without imputation. Associations between vaccination uptake and demographic characteristics, HIV-related clinical parameters, healthcare behaviours, and vaccine-related perceptions were explored through univariate analysis.

Variables considered conceptually appropriate as potential independent predictors of vaccination uptake and showing an association with vaccination status in univariate analyses (*p* < 0.05), together with clinically relevant covariates selected a priori based on the existing literature, were considered for inclusion in the multivariable binary logistic regression model, with vaccination status as the dependent variable. Reported reasons for non-vaccination were analyzed separately as self-reported barriers and were not considered candidate predictors for the multivariable model because they directly describe reasons underlying the vaccination behaviour represented by the dependent variable. Educational level, CD4 + T-cell count category, and beliefs regarding the role of vaccination in preventing hospitalization met the inclusion criteria and were included in the final model. Results were expressed as odds ratios (ORs) with corresponding 95% confidence intervals (95% CI). In addition, a composite variable describing pandemic-related negative impact was developed. This variable incorporated self-reported avoidance of medical visits during the COVID-19 pandemic, perceived difficulties in accessing healthcare services, and a perceived negative influence of the pandemic on overall health status. Participants reporting at least one adverse pandemic-related experience were classified as having experienced a pandemic-related negative impact. Associations between this variable, vaccination uptake, preventive attitudes, and information-seeking behaviour were subsequently evaluated.

All statistical tests were two-sided, and a *p*-value < 0.05 was considered statistically significant.

## 3. Results

A total of 203 people living with HIV were included in the study, comprising 151 men (74.4%) and 52 women (25.6%). The median age was 39 years (IQR 34–50). Most participants resided in urban areas (77.3%) and completed at least high school education (76.8%). Regarding occupational status, 43.3% were employed, while 36.9% were retired. Most participants (45.3%) had been diagnosed with HIV for more than 10 years and had received antiretroviral therapy for more than 10 years (42.9%). An undetectable HIV viral load was documented in 67.5% of participants, while 63.1% had a CD4+ T-cell count >500 cells/mm^3^. High adherence to antiretroviral therapy (>95%) was reported by participants in 85.2% of cases (Table 1).

To evaluate overall vaccination uptake, a composite variable was created based on receipt of at least one vaccine assessed in the questionnaire during the previous 3 years, including both routinely recommended vaccines for PLWH and vaccines indicated according to individual risk factors (including influenza, pneumococcal, meningococcal, hepatitis A, hepatitis B, varicella, HPV, COVID-19, or other vaccines). Participants who reported receiving at least one dose of any of the assessed vaccines were classified as “vaccinated,” while those who reported receiving none were classified as “not vaccinated.” Among the 203 participants, 79 (38.9%) were classified as vaccinated, while 124 (61.1%) were classified as not vaccinated (Table 2).

When vaccine-specific uptake was evaluated, influenza vaccination was the most frequently reported vaccine (27.1%), followed by COVID-19 vaccination (12.3%) and hepatitis B vaccination (9.4%). Uptake of pneumococcal, hepatitis A, HPV, meningococcal and varicella vaccines remained low, ranging from 0.5% to 2.5%. Among the 79 vaccinated participants, 51 (25.1% of the total cohort) reported receiving one vaccine, while 28 (13.8% of the total cohort) reported receiving two or more vaccines (Table 2).

Given the broad definition of the composite vaccination outcome, an additional vaccine-specific analysis was performed for influenza and COVID-19 vaccination. Overall, 55 participants (27.1%) reported influenza vaccination and 25 (12.3%) reported COVID-19 vaccination during the previous three years: 69 participants (34%) had received at least one of these two vaccines while 11 (5.4%) had received both. Influenza vaccination was significantly associated with the belief that vaccines help prevent hospitalization (76.4% among influenza-vaccinated vs. 52% among non-influenza-vaccinated participants, *p* = 0.003). COVID-19 vaccination was significantly associated with undetectable HIV viral load (94.7% among COVID-19 vaccinated vs. 72.1% among non-COVID-19 vaccinated participants, Fisher’s exact *p* = 0.048). No other significant associations were identified in the vaccine-specific analyses (Table 3).

Vaccination uptake was further evaluated in relation to demographic and HIV-related clinical characteristics. Educational level was significantly associated with vaccination uptake (*p* = 0.048), with university-level education being more frequent among vaccinated than non-vaccinated participants (39.2% vs. 26.0%). Vaccination uptake was also significantly associated with CD4+ T-cell count category (*p* = 0.023), and CD4+ T-cell counts >500 cells/mm3 were more frequent among vaccinated participants than among non-vaccinated participants (78.3% vs. 62.2%, respectively) (Table 4). In contrast, no significant associations were observed with sex, age, HIV viral load category, or estimated ART adherence. Regarding vaccine hesitancy, the most frequently reported reason for non-vaccination was the perception that vaccination was unnecessary (36.5%), followed by lack of physician recommendation (20.7%), fear of adverse effects (18.7%), lack of trust in vaccines (15.3%), and lack of time (7.4%). Comparisons according to vaccination status showed significant differences for the perception that vaccination was unnecessary (*p* = 0.003), fear of adverse effects (*p* < 0.001), and lack of trust in vaccines (*p* = 0.001), whereas no significant differences were observed for lack of physician’s recommendation or lack of time (*p* = 0.645) (Table 4).

Most participants agreed that PLWH are more vulnerable to infections (68.5%), while 15.8% disagreed and 15.8% expressed a neutral opinion. No statistically significant association was observed between vaccination uptake and the perception that PLWH are more vulnerable to infections (*p* = 0.558). Regarding perceived vaccine benefits, 58.1% of participants agreed that vaccines help prevent hospitalization, while 23.3% expressed a neutral opinion and 18.7% disagreed. A statistically significant association was observed between this belief and vaccination uptake (*p* = 0.006), with vaccinated participants more frequently agreeing that vaccines help prevent hospitalization than non-vaccinated participants (72.2% vs. 50.0%, respectively) (Table 4).

Regarding healthcare information sources, infectious diseases specialists represented the most frequently reported source of medical information, either alone or in combination with other sources. Family physicians and internet/social media platforms were also commonly reported. More than half of the participants (54.2%) reported that they would like infectious disease specialists to provide additional information regarding the prevention of acute infections, while 37.9% did not express this need and 7.9% were uncertain.

Only 24.6% of participants reported continued use of protective masks in crowded spaces or public transportation, whereas 75.4% reported not using masks in these settings.

Pandemic-related healthcare experiences are summarized in Table 5. Overall, 19.2% of participants reported avoiding hospital visits during the COVID-19 pandemic, 35.5% reported more difficult access to healthcare services, and 5.9% experienced difficulties obtaining antiretroviral therapy. Confirmed COVID-19 infection was reported by 34.0% of participants, while only 3% required hospitalization. More than half of the participants (52.7%) met the criteria for the composite pandemic-related negative impact variable. When these variables were evaluated according to vaccination status, confirmed COVID-19 infection was significantly more frequent among vaccinated than non-vaccinated participants (43.0% vs. 28.2%, respectively; *p* = 0.030). In contrast, the pandemic-related negative impact composite did not differ significantly according to vaccination status (58.2% vs. 49.2%, respectively; *p* = 0.209). No statistically significant differences according to vaccination status were observed for the other pandemic-related variables presented in Table 5.

### Factors Independently Associated with Vaccination Uptake

Variables included in the multivariable logistic regression model were selected based on clinical relevance and statistically significant findings identified in the univariate analyses. The final model included university-level education, CD4+ T-cell count > 500 cells/mm^3^, and the belief that vaccination helps prevent hospitalization. Adjusted odds ratios (ORs), 95% confidence intervals (95% CIs), and *p*-values were calculated, and the results are presented in Table 6.

Multivariable logistic regression analysis demonstrated that positive vaccine-related beliefs and better immunological status remained independently associated with vaccination uptake. Participants who agreed that vaccines help prevent hospitalization had significantly lower odds of being non-vaccinated (OR = 0.454, 95% CI: 0.235–0.876, *p* = 0.019). Similarly, participants with CD4+ T-cell counts above 500 cells/mm^3^ had significantly lower odds of being non-vaccinated (OR = 0.448, 95% CI: 0.232–0.867, *p* = 0.017). In contrast, university-level education was not independently associated with vaccination uptake after adjustment (OR = 0.761, 95% CI: 0.394–1.469, *p* = 0.416) (Table 6).

This variable combined responses regarding avoidance of hospital visits during the pandemic, perceived difficulty accessing healthcare services, and self-reported negative effects on general health status. Overall, 52.7% of participants met the criteria for pandemic-related negative impact.

The pandemic-related negative impact composite was reported by 58.2% of vaccinated participants and 49.2% of non-vaccinated participants; however, this difference was not statistically significant (*p* = 0.209) (Table 5). In contrast, a confirmed history of COVID-19 infection was significantly more frequent among vaccinated than non-vaccinated participants (43.0% vs. 28.2%, respectively; *p* = 0.030) (Table 5).

No significant association was observed between pandemic-related negative impact and the belief that vaccines help prevent hospitalization (*p* = 0.516) (Table 7).

A statistically significant association was observed between pandemic-related negative impact and the desire for additional information from infectious diseases specialists regarding the prevention of acute infections (*p* = 0.011). Participants reporting pandemic-related negative experiences more frequently expressed the need for additional medical information than those who did not report a pandemic-related impact (62.6% vs. 44.8%, respectively) (Table 7).

## 4. Discussion

Our study provides insight into vaccine-related perceptions, vaccine hesitancy reasons, healthcare informational needs, and pandemic-related experiences among PLWH receiving long-term HIV care. Our findings demonstrate low vaccination uptake and identify vaccine-related beliefs and higher CD4+ T-cell counts as important factors associated with vaccination uptake, while pandemic-related experiences were associated with selected preventive healthcare behaviours and information needs. Among the 203 participants included, the study population consisted of middle-aged men, in line with findings from previous studies [27]. Most participants resided in urban areas and had at least a high school education, a demographic profile consistent with that reported in previous studies involving PLWH [28]. The study population comprised similar proportions of employed and retired individuals.

An important strength of our cohort is that half of the participants had been living with HIV for more than 10 years and had received antiretroviral therapy for a similar period. This indicates that most participants were not newly diagnosed or recently initiated on treatment but had extensive experience with long-term HIV care. The high proportion of participants with long-standing HIV infection and prolonged exposure to antiretroviral therapy is consistent with recent epidemiological data describing the maturation of the Romanian HIV cohort and the increasing number of PLWH requiring long-term multidisciplinary care [29]. Previous Romanian studies have also highlighted specific clinical and biological factors associated with HIV-related outcomes, including mother-to-child HIV transmission, further emphasizing the complexity of long-term HIV care across different patient populations [30]. Consequently, they had repeated interactions with healthcare providers, were familiar with the healthcare system, and had multiple opportunities to receive counselling regarding preventive measures, including vaccination. The good adherence to ART reported in our study indicates that the study population was well engaged in HIV care. This suggests that participants were familiar with the healthcare system and had regular contact with infectious diseases specialists, providing multiple opportunities for preventive interventions, including vaccination, consistent with the concept of the HIV care continuum [30].

An important finding of the present study relates to participants’ perceptions regarding infection risk and vaccination. Although most respondents recognized the increased vulnerability of PLWH to infectious diseases, and more than half believed that vaccination helps prevent hospitalization, overall vaccination uptake remained low. This discrepancy suggests that awareness alone may be insufficient to translate into preventive action and highlights the importance of physician recommendation, vaccine confidence, and effective healthcare communication [31]. Furthermore, a substantial proportion of participants either disagreed or expressed uncertainty regarding the increased susceptibility of PLWH to infections, indicating that important knowledge gaps may persist even among individuals receiving long-term HIV care [32].

Vaccination uptake remained low, with only 38.9% of participants reporting receipt of at least one vaccine assessed during the previous three years. Influenza vaccination represented the most frequently reported vaccine in our cohort, whereas uptake of several other recommended vaccines, including pneumococcal, meningococcal, HPV and varicella vaccines, remained extremely low. Similar findings have been reported in other studies involving PLWH, where vaccination coverage remains suboptimal despite clear guideline recommendations. The recent expansion of reimbursement programs for several vaccines recommended for PLWH in Romania may further support equitable access to vaccination, although additional efforts are still needed to improve vaccine coverage in this population.

One of the most important findings of the present study was the significant association between positive vaccine-related beliefs and vaccination uptake. Participants who believed that vaccines help prevent hospitalization were significantly more likely to report receiving at least one vaccine within the previous three years [33].

Higher CD4+ T-cell counts were also independently associated with vaccination uptake in our analysis. However, this association should not necessarily be interpreted as evidence of a direct biological effect of immunological status on vaccination behaviour. Higher CD4+ T-cell counts may instead reflect sustained engagement in HIV care, regular medical follow-up, long-term adherence to antiretroviral therapy, and greater participation in preventive healthcare activities. Another explanation is that healthcare providers may have been more likely to recommend vaccination to patients with higher CD4+ T-cell counts, potentially reflecting perceptions regarding the expected benefit or effectiveness of vaccination in individuals with better immune status. However, our study did not collect data linking individual physician recommendations to CD4+ T-cell counts; therefore, this possibility cannot be directly evaluated and should be considered hypothesis- generating. Thus, the observed association may partly represent broader healthcare engagement rather than a direct effect of CD4+ T-cell count on vaccine acceptance. Educational level was also associated with vaccination uptake in the univariate analysis, with university-level education being more frequent among vaccinated participants. However, this association was no longer statistically significant after adjustment in the multivariable model. This finding suggests that the relationship between education and vaccination behaviour may be partly explained by other factors, including healthcare engagement and vaccine-related beliefs, rather than by educational attainment alone. Taken together, the multivariable findings suggest that vaccination uptake in this cohort was associated with both healthcare-related factors, reflected by higher CD4+ T-cell counts, and individual perceptions regarding the benefits of vaccination.

The analysis of vaccine hesitancy provided additional insights into the barriers to vaccination in our cohort. The most frequently reported reasons for non-vaccination were the perception that vaccination was unnecessary, lack of physician recommendation, concerns regarding adverse effects, and lack of trust in vaccines. These findings are consistent with previous studies identifying perceived lack of need, safety concerns, and insufficient healthcare provider recommendations as important barriers to vaccination among PLWH [34,35,36]. The relevance of healthcare providers was further supported by our finding that infectious diseases specialists represented the most frequently reported source of medical information and that more than half of the participants expressed a desire to receive additional information regarding infection prevention from these specialists. Similar studies have reported that healthcare-provider recommendation and trust in HIV care providers are important determinants of vaccine acceptance among PLWH [28,34,36]. These findings support the integration of vaccination assessment and counselling into routine HIV care, in accordance with current EACS recommendations [12].

The overall vaccination uptake observed in our cohort was low, with 38.9% of participants reporting receipt of at least one of the vaccines assessed during the previous three years. Influenza vaccination was the most frequently reported, whereas uptake of pneumococcal, meningococcal, HPV, and varicella vaccines remained particularly low. Vaccination coverage in our cohort was lower than that reported in several other PLWH populations. In a Danish cohort of 1031 virologically suppressed PLWH, 53.2% had received at least one influenza vaccination and 31% at least one pneumococcal vaccination during the respective study periods [37]. Similarly, a recent study involving 791 PLWH reported influenza and pneumococcal vaccination coverage of 68% and 37.8%, respectively, while 89.1% had received at least one dose of a COVID-19 vaccine [38]. In a large cohort of 22,058 PLWH, Hechter et al. reported 90.5% coverage with the primary COVID-19 vaccination series [39]. The clinical relevance of improving vaccination coverage in PLWH is further supported by recent evidence from Horberg et al., who reported a higher risk of post-COVID conditions among PLWH compared with people without HIV and found that COVID-19 vaccination reduced the risk of acute-persistent post-COVID conditions [40].

These findings illustrate the substantial heterogeneity in vaccination coverage and vaccine-related outcomes among PLWH across different healthcare settings and countries.

Differences in national vaccination policies, reimbursement mechanisms, healthcare-provider practices, study periods, and characteristics of the populations studied may partly explain the variability in uptake across cohorts.

An important objective of the present study was to explore pandemic-related healthcare experiences among PLWH and their potential relationship with preventive healthcare behaviours. Although these variables were not originally designed to evaluate vaccination uptake, they provide valuable insight into the experiences of a population characterized by long-term engagement in HIV care. Most participants had been living with HIV and were receiving antiretroviral therapy for more than 10 years. Consequently, many had previous experiences navigating chronic disease management, healthcare access challenges, and prolonged interactions with healthcare providers. This context may be particularly relevant when interpreting their experiences during the COVID-19 pandemic, as PLWH represent a population requiring sustained engagement with healthcare systems and continuity of HIV care [41].

Approximately one-third of the participants reported more difficult access to medical services, while nearly one-fifth reported avoiding hospital visits for medical follow-up during the pandemic. To better characterize the overall effect of the pandemic, a composite variable reflecting pandemic-related negative impact was created by combining pandemic-associated healthcare disruption and self-reported negative health effects.

More than half of participants experienced at least one negative pandemic-related effect. These findings should also be interpreted in the context of long-standing HIV history observed in our cohort. This long-standing engagement of many participants with HIV care may have influenced how healthcare disruption, vulnerability, and risk communication were experienced during the pandemic [42]. However, the composite measure of pandemic-related negative impact was not significantly associated with vaccination status. Interestingly, participants with a history of confirmed COVID-19 infection were more frequently vaccinated than those without a confirmed infection. Although causality cannot be inferred from the cross-sectional design, previous personal experience with COVID-19 may have influenced subsequent preventive behaviour or may reflect greater engagement with healthcare services.

Pandemic-related negative experiences were not significantly associated with the belief that vaccines help prevent hospitalization. Together with the absence of a significant association between the pandemic-related negative impact composite and vaccination status, this finding suggests that the relationship between pandemic experiences, vaccine-related beliefs, and preventive behaviour may be complex and should not be interpreted as causal.

Another important finding was the significant association between pandemic-related negative impact and the desire for additional medical information from infectious diseases specialists. Participants reporting negative pandemic-related experiences more frequently expressed the need for additional information regarding prevention of acute infections. This finding highlights the increased informational and educational needs that may emerge following periods of healthcare disruption and public health crisis [43].

The present study has several strengths. First, it evaluates multiple dimensions of preventive healthcare behaviour among PLWH, including vaccination uptake, vaccine hesitancy, preventive beliefs, healthcare information source, and pandemic-related healthcare experiences. Second, the inclusion of multivariable logistic regression analysis strengthened the evaluation of independent predictors associated with vaccination uptake. Third, the study addresses a clinically relevant topic in the post-pandemic period, when the long-term impact of COVID-19 on preventive healthcare behaviour remains insufficiently understood. A particular strength of the study is the simultaneous evaluation of vaccination uptake, vaccine-related perceptions, healthcare information sources, and pandemic-related experiences within a well-characterized cohort of PLWH.

Several limitations also should be acknowledged. The study was based on self-reported questionnaire data, which may introduce recall bias and subjective interpretation of responses. The cross-sectional design does not allow causal inference regarding the relationship between pandemic-related experiences, vaccine perceptions, and vaccine behaviour. In addition, this study was conducted in a single-centre population, potentially limiting the applicability of the results to broader PLWH populations. Although the study centre provides care to a large population of more than 4000 PLWH, aggregate demographic data for the entire clinic population were not available for comparison; therefore, the representativeness of the study sample relative to the overall clinic population could not be formally assessed. In addition, although complete questionnaire data were required for inclusion, some clinical variables derived from routine medical records, including the last documented viral load, CD4+ T-cell count, and ART adherence, were unavailable for a small proportion of participants. Analyses involving these variables were therefore based on available cases. Variability in the availability of clinical information in routine medical records and the use of available-case analysis for variables with missing observations may have introduced selection bias and should be considered when interpreting associations involving these variables. Some questionnaire variables also required recategorization or grouping for statistical analysis due to multiple overlapping responses. Furthermore, the composite vaccination variable did not distinguish between the number of vaccines received or adherence to all vaccine recommendations and therefore should be interpreted as a measure of overall vaccination uptake rather than complete vaccination coverage. The questionnaire was specifically developed for the purposes of this study and did not undergo formal psychometric validation. In addition, health literacy and vaccine hesitancy were not assessed using validated standardized instruments. Although the study-specific questionnaire included items addressing vaccine-related perceptions, reasons for non-vaccination, and sources of health information, these measures do not provide a standardized assessment of health literacy or vaccine hesitancy. Future studies should incorporate validated instruments to better characterize these constructs and their relationship with vaccination uptake among PLWH.

Despite these limitations, the present findings provide important insights into preventive healthcare behaviours among PLWH in the post-COVID-19 period. The study suggests that preventive healthcare behaviour among PLWH was associated not only with clinical status, but also with healthcare-related experiences, physician communication, and preventive health perceptions.

## 5. Conclusions

Vaccination uptake among PLWH in our cohort remained suboptimal, despite regular engagement with HIV care and a relatively high awareness of infection-related vulnerability. Vaccine-related beliefs and CD4+ T-cell count were independently associated with vaccination uptake, while lack of perceived need, absence of physician recommendation, concerns regarding adverse effects, and lack of trust emerged as important barriers. These findings highlight the significant role of healthcare providers, particularly infectious diseases specialists, in promoting preventive vaccination among PLWH.

From a public health and policy perspective, our findings support the implementation of systematic vaccination-status assessment during routine HIV follow-up visits, coupled with active, individualized vaccine recommendations from healthcare professionals. Integration of vaccination reminders and standardized vaccination checklists into HIV care pathways could facilitate identification of missed vaccination opportunities. Policymakers should also prioritize targeted educational interventions addressing vaccine safety, effectiveness, and perceived necessity, while ensuring that healthcare professionals have access to clear and updated vaccination guidance for PLWH. Strengthening coordination between infectious diseases specialists, primary care providers, and vaccination services may further improve continuity of preventive care. These measures could provide a practical framework for improving vaccination uptake among PLWH, although their effectiveness should be evaluated in larger, multicentre studies.

## Figures and Tables

**Table 1:** Sociodemographic and clinical characteristics of the study population.

Patients (n = 203)	n (%)
**Sex**	
Male	151 (74.4)
Female	52 (25.6)
**Age, median (IQR)**	
39 (IQR: 34–50)	
**Educational level**	
No formal education	6 (3.0)
Primary education	8 (3.9)
Secondary education	32 (15.8)
High School	93 (45.8)
University degree	63 (31)
Not reported	1 (0.5)
**Residence**	
Urban	157 (77.3)
Rural	46 (22.7)
**Occupational status**	
Employed	88 (43.3)
Retired	75 (36.9)
Unemployed	35 (17.2)
Student	5 (2.0)
**Time since HIV diagnosis**	
<1 year	18 (8.9)
1–5 years	56 (27.6)
6–10 years	37 (18.2)
>10 years	92 (45.3)
**Time since ART initiation**	
<1 year	20 (9.9)
1–5 years	58 (28.6)
6–10 years	38 (18.7)
>10 years	87 (42.9)
**Last documented viral load**	
Undetectable	137 (67.5)
Detectable < 1000 copies/mL	37 (18.2)
Detectable ≥ 1000 copies/mL	10 (4.9)
Missing data	19 (9.4)
**Last CD4 count**	
<200 cells/mm^3^	13 (6.4)
200–350 cells/mm^3^	16 (7.9)
351–500 cells/mm^3^	31 (15.3)
>500 cells/mm^3^	128 (63.1)
Missing data	15 (7.4)
**ART adherence**	
<80% (low)	5 (2.5)
80–94% (good)	9 (4.4)
>95% (very good)	173 (85.2)
Missing data	16 (7.9)

Abbreviations: ART = antiretroviral therapy; IQR = interquartile range.

**Table 2:** Overall vaccination status, self-reported vaccination uptake by vaccine type, and number of vaccines received during the previous three years.

Vaccination Variable	n (%)
**Overall vaccination status**	
Vaccinated	79 (38.9)
Not vaccinated	124 (61.1)
**Number of vaccines received**	
One vaccine	51 (25.1)
Two or more vaccines	28 (13.8)
**Vaccine type**	
Influenza	55 (27.1)
COVID-19	25 (12.3)
Hepatitis B	19 (9.4)
Hepatitis A	5 (2.5)
Pneumococcal	4 (2.0)
HPV	4 (2.0)
Meningococcal	1 (0.5)
Varicella	1 (0.5)
Other	1 (0.5)

**Table 3:** Vaccine-specific analysis of factors associated with influenza and COVID-19 vaccination uptake.

Characteristic	Influenza Vaccinated n = 55	Influenza Not Vaccinated n = 148	*p*-Value	COVID-19 Vaccinated. n = 25	COVID-19 Not Vaccinated n = 178	*p*-Value
Male sex	40 (72.7%)	111 (75%)	0.882	19 (76%)	132 (74.2%)	1.000
University education	22 (40%)	41 (27.9%)	0.138	9 (36%)	54 (30.5%)	0.746
CD4+ T-cell count > 500 cells/mm^3^	39 (79.6%)	89 (64%)	0.067	13 (68.4%)	115 (68%)	1.000
Undetectable viral load	37 (77.1%)	100 (73.5%)	0.770	18 (94.7%)	119 (72.1%)	0.048
ART adherence ≥ 95%	47 (97.9%)	126 (90.6%)	0.121	19 (100%)	154 (91.7%)	0.368
PLWH are more vulnerable-agree	38 (69.1%)	101 (68.2%)	1.000	17 (68%)	122 (68.5%)	1.000
Vaccines help prevent hospitalization-agree	42 (76.4%)	77 (52%)	0.003	19 (76%)	100(56.2%)	0.095

**Note:** percentages for CD4+ T-cell count, viral load, and ART adherence were calculated among participants with available data for the respective variable, rather than using the total N for each group.

**Table 4:** Demographic and clinical characteristics, reported barriers to vaccination, and preventive health-related perceptions according to vaccination status.

Variable	Vaccinated n (%)	Non-Vaccinated n (%)	*p*-Value
**Demographic and clinical characteristics**			
Sex			0.461
Male	61 (77.2)	90 (72.6)	
Female	18 (22.8)	34 (27.4)	
Age, median (IQR)	41 (34.5–52)	38 (34–49)	0.130
University-level education	31 (39.2)	32 (25.8)	0.048
**HIV viral load category**			0.498
Undetectable	54 (79.4)	83 (71.6)	
Detectable < 1000 copies/mL	11 (16.2)	26 (22.4)	
Detectable ≥ 1000 copies/mL	3 (4.4)	7 (6.0)	
**CD4+ T-cell count > 500 cells/mm^3^**	54 (78.3)	74 (62.2)	0.023
**Estimated ART adherence**			0.056
≥95% (very good)	67 (98.5)	106 (89.1)	
80–94% (good)	1 (1.5)	8 (6.7)	
<80% (low)	0 (0.0)	5 (4.2)	
**Reported reasons for non-vaccination**			
Vaccination perceived as unnecessary	19 (24.1)	55 (44.4)	0.003
Lack of physician recommendation	17 (21.5)	25 (20.2)	0.816
Fear of adverse effects	5 (6.3)	33 (26.6)	<0.001
Lack of trust in vaccines	4 (5.1)	27 (21.8)	0.001
Lack of time	5 (6.3)	10 (8.1)	0.645
**Vaccine-related perceptions**			
**PLWH are more vulnerable to infections**			0.558
Agree	53 (67.1)	86 (69.4)	
Neutral	11 (13.9)	21 (16.9)	
Disagree	15 (19.0)	17 (13.7)	
**Vaccines help prevent hospitalization**			0.006
Agree	57 (72.2)	62 (50.0)	
Neutral	11 (13.9)	36 (29.0)	
Disagree	11 (13.9)	26 (21.0)	
**Need for additional preventive information from infectious diseases specialists**			0.621
Yes	41 (51.9)	69 (55.6)	
No	30 (38.0)	47 (37.9)	
Uncertain	8 (10.1)	8 (6.5)	

Statistical comparisons between vaccinated and non-vaccinated participants were performed using Pearson’s chi-square test or Fisher’s exact test, as appropriate, for categorical variables and the Mann–Whitney U test for age. Analyses involving clinical variables with missing observations were based on available cases only, without imputation. A *p*-value <0.05 was considered statistically significant. Multiple responses were permitted for reported reasons for non-vaccination; therefore, percentages do not sum to 100%. Each reported reason for non-vaccination was treated as a separate binary variable, and the corresponding *p*-value represents the comparison of that individual reason according to vaccination status. Abbreviations: PLWH, people living with HIV; ART, antiretroviral therapy; IQR, interquartile range.

**Table 5:** Impact of the COVID-19 pandemic on healthcare access and preventive behaviours among people living with HIV.

Variable	Vaccinated n (%)	Non-Vaccinated n (%)	*p*-Value
Continued mask use in crowded spaces/public transport	17 (21.5)	33 (26.6)	0.411
Avoided hospital visits during pandemic	15 (19.0)	24 (19.4)	0.948
More difficult healthcare access during pandemic	33 (41.8)	39 (31.5)	0.134
Difficulties obtaining ART	7 (8.9)	5 (4.0)	0.221 *
Confirmed COVID-19 infection	34 (43.0)	35 (28.2)	0.030
COVID-19-related hospitalization	3 (3.8)	3 (2.4)	0.680 *
Negative impact on general health	15 (19.0)	21 (16.9)	0.709
Negative impact on physicians’ communication	7 (8.9)	10 (8.1)	0.842
Pandemic-related negative impact composite	46 (58.2)	61 (49.2)	0.209

Statistical comparisons between vaccinated and non-vaccinated participants were performed using Pearson’s chi-square test or Fisher’s exact test, as appropriate. * Fisher’s exact test was used for difficulties obtaining ART and COVID-19-related hospitalization because of small expected cell counts. A *p*-value < 0.05 was considered statistically significant. The pandemic-related negative impact composite was defined as reporting at least one of the following: avoidance of medical visits during the COVID-19 pandemic, perceived difficulties in accessing healthcare services, or a perceived negative influence of the pandemic on overall health status. Abbreviations: ART, antiretroviral therapy.

**Table 6:** Multivariable logistic regression analysis of factors associated with vaccination uptake among people living with HIV.

Variable	OR	95% CI	*p* Value
University-level education	0.761	0.394–1.469	0.416
Positive belief that vaccines prevent hospitalization	0.454	0.235–0.876	0.019
CD4 count > 500 cells/mm^3^	0.448	0.232–0.867	0.017

**Table 7:** Associations between pandemic-related negative impact and vaccination- and preventive health-related factors.

Outcome	Pandemic-Related Negative Impact, n (%) (n = 107)	No pandemic-Related Negative Impact, n (%) (n = 96)	*p*-Value
Vaccinated	46 (43.0)	33 (34.4)	0.209
Belief that vaccines help prevent hospitalization: Agree	65 (60.7)	54 (56.3)	0.516
Desire for additional information from infectious diseases specialists: Yes	67 (62.6)	43 (44.8)	0.011

Statistical comparisons were performed using Pearson’s chi-square test. A *p*-value <0.05 was considered statistically significant.

## Data Availability

The original contributions presented in this study are included in the article/Supplementary Materials. Further inquiries can be directed to the corresponding authors.

## Supplementary Materials

The following supporting information can be downloaded at: https://www.mdpi.com/article/10.3390/vaccines14090751/s1, File S1: Questionnaire on the Impact of Acute Infectious Diseases.
