# Supplementary material for: Vaccination Uptake and Pandemic-Related Healthcare Experiences Among People Living with HIV in Romania: A Cross-Sectional Study
Source: Vaccines (Basel). 2026 Aug 28;14(9):751. doi: 10.3390/vaccines14090751 (PMC13611687; doi:10.3390/vaccines14090751)
Supplement: Supplementary file 1 [file vaccines-14-00751-s001.zip › vaccines-4474864-supplementary.pdf]

No. [______________]

Questionnaire on the Impact of Acute Infectious Diseases

Part 1 - To be completed by the patient

1. Sex: ☐Male / ☐Female

2. Age: ☐years

3. Area of residence: ☐Urban / ☐Rural

4. Level of education, highest level completed:

☐No formal education / ☐Primary school / ☐Lower secondary school / ☐High school / ☐University, Master's degree

5. Occupation:

☐Employed / ☐Unemployed / ☐Retired / ☐Student / ☐No occupation

6. Time since HIV diagnosis:

☐<1 year / ☐1-5 years / ☐6-10 years / ☐>10 years

7. Time since initiation of HIV therapy:

☐<1 year / ☐1-5 years / ☐6-10 years / ☐>10 years

8. During the past year, have you had acute infections? (check all that apply)

- respiratory infections? ☐YES / ☐ NO; if YES, approximate number [__________]
- gastrointestinal infections? ☐YES / ☐NO; if YES, approximate number [___________]
- urinary infections? ☐YES / ☐NO; if YES, approximate number [__________]
- other infections? ☐YES / ☐NO; if YES, specify the type of infection(s) and approximate number: [ ]

9. During the past year, have you been diagnosed with:

- Influenza ☐YES / ☐NO
- COVID-19 ☐YES / ☐NO
- Acute pneumonia ☐YES / ☐NO
- Acute otitis ☐YES / ☐NO
- Acute sinusitis ☐ YES / ☐NO
- Acute gastroenterocolitis ☐YES / ☐NO
- Urinary tract infection ☐YES / ☐NO

No. [______________]

Questionnaire on the Impact of Acute Infectious Diseases

10. During the past year, how many hospitalizations for acute infections have you had?

☐0 / ☐1 / ☐2-3 / ☐≥4

11. When you have an acute infection, whom do you usually consult?

☐Family physician

☐Infectious Diseases physician

☐Emergency Department (CPU/UPU)

☐Another specialist physician

☐Pharmacy, where I ask for treatment recommendations

☐I self-treat

12. During the past year, how many times have you taken antibiotics?

☐0 / ☐1-2 / ☐3-5 / ☐>5 / ☐I don't know

13. The antibiotics were prescribed/recommended by:

☐Infectious Diseases physician

☐Family physician

☐Another specialist physician

☐Pharmacist

☐Self-medication

14. How do you consider the following statements?

Statement	True	False	Don't know
Antibiotics treat only bacterial infections	☐	☐	☐
Antibiotics are useful for respiratory viral infections	☐	☐	☐
It is acceptable to stop antibiotics when I feel better	☐	☐	☐
Frequent use of antibiotics can lead to resistance	☐	☐	☐
People with HIV need to take antibiotics more often than others	☐	☐	☐
If I take antibiotics preventively, I can avoid becoming ill	☐	☐	☐

15. During the past 3 years, have you received any of the following vaccines?

• influenza vaccine ☐Yes / ☐No

• COVID-19 vaccine ☐Yes / ☐No

• pneumococcal vaccine ☐Yes / ☐No

• meningococcal vaccine ☐Yes / ☐No

• hepatitis B vaccine ☐Yes / ☐No

No. [______________]

Questionnaire on the Impact of Acute Infectious Diseases

• hepatitis A vaccine ☐ Yes / ☐No

• varicella vaccine ☐Yes / ☐N

• HPV vaccine ☐Yes / ☐No

• other vaccine ☐Yes / ☐No; if YES, which vaccine? [____]

☐I have not received any vaccine

16. If you have NOT been vaccinated, the main reasons are (check all that apply):

☐Fear of adverse reactions

☐I do not consider it necessary

☐Lack of recommendation from my physician

☐Lack of time

☐Difficult access

☐I do not trust vaccines

☐Other reason: [____]

17. How do you rate the following statements?

Statement	Agree	Neutral	Disagree
People with HIV are more vulnerable to infections	☐	☐	☐
Vaccines help prevent hospitalizations	☐	☐	☐
Information about infections reduces my anxiety	☐	☐	☐
The recommendations of my Infectious Diseases physician are sufficient for my protection	☐	☐	☐

18. Do you agree or disagree with the following statements?

Statement	Agree	Disagree	Don't know
Antiretroviral therapy completely protects me against other infections	☐	☐	☐
If I have HIV, antibiotics are less effective	☐	☐	☐
Vaccines can reactivate HIV	☐	☐	☐
It is better to treat infections when they occur than to get vaccinated	☐	☐	☐

20. Where do you most often obtain health information?

☐Infectious Diseases physician

☐Family physician

No. [______________]

Questionnaire on the Impact of Acute Infectious Diseases

☐Another specialist physician

☐Internet / Social media

☐Family / Friends

☐Other: [____]

21. Would you like your Infectious Diseases physician to provide you with more information about preventing acute infections?

☐Yes / ☐ No / ☐ Not sure

22. Do you currently wear a protective mask in crowded spaces or on public transport?

☐Yes / ☐No

23. During the COVID-19 pandemic, did you avoid coming to the hospital for follow-up visits?

☐Yes / ☐No / ☐I don't remember

24. Did you have difficulties obtaining antiretroviral medication during the COVID-19 pandemic?

☐Yes / ☐No / ☐Not applicable, I was not taking antiretroviral therapy at that time

25. How would you rate access to healthcare services during the pandemic compared with the present?

☐Much more difficult / ☐Slightly more difficult / ☐The same / ☐Easier

26. If you needed urgent medical care during the pandemic, were you able to obtain it quickly?

☐Yes / ☐No / ☐I did not need urgent care

27. During the pandemic, did you have a confirmed COVID-19 infection?

☐Yes, once / ☐Yes, more than once / ☐No / ☐I don't know

28. If YES, was hospitalization required?

☐Yes / ☐No / ☐Not applicable

29. Did the pandemic affect your overall health?

☐Yes, significantly / ☐ Yes, to some extent / ☐Not much / ☐Not at all

30. Did the pandemic affect your relationship with physicians and communication regarding your health?

☐Yes, negatively / ☐No, it remained the same / ☐Yes, positively (more support)

No. [______________]

Questionnaire on the Impact of Acute Infectious Diseases

Part 2 - To be completed by the physician

1. Time since HIV diagnosis:

☐<1 year / ☐1-5 years / ☐6-10 years / ☐>10 years

2. Time since initiation of HIV therapy:

☐<1 year / ☐1-5 years / ☐6-10 years / ☐>10 years

3. Most recent HIV viral load (HIV-RNA):

☐Undetectable / ☐Detectable <1000 copies/mL / ☐Detectable >1000 copies/mL

4. Most recent CD4 count:

☐<200 / ☐200-350 / ☐351-500 / ☐>500

5. Relevant co-infections / comorbidities (check all that apply):

- HBV
- HCV
- TB
- Cardiovascular disease
- Obesity
- Diabetes
- COPD
- Malignancies

Other: [____]

6. History of opportunistic infections: ☐Yes / ☐No

7. Current ART regimen: [____]

8. Physician-estimated ART adherence:

☐95% (very good) / ☐80-94% (partial) / ☐<80% (poor) / ☐Unknown

9. ART interruptions during the past year:

☐Yes / ☐No

10. Relevant details for interpreting the patient's responses:

____

____
